# Staff exchange within and between nursing homes in The Netherlands and potential implications for MRSA transmission

**DOI:** 10.1017/S0950268816002831

**Published:** 2016-12-05

**Authors:** R. D. VAN GAALEN, H. A. HOPMAN, A. HAENEN, C. VAN DEN DOOL

**Affiliations:** Centre for Infectious Disease Control, National Institute for Public Health and the Environment (RIVM), Bilthoven, The Netherlands

**Keywords:** Disease transmission, methicillin – *S. aureus* resistant to (MRSA), nursing homes, staff exchange

## Abstract

A recent countrywide MRSA *spa*-type 1081 outbreak in The Netherlands predominantly affected nursing homes, generating questions on how infection spreads within and between nursing homes despite a low national prevalence. Since the transfer of residents between nursing homes is uncommon in The Netherlands, we hypothesized that staff exchange plays an important role in transmission. This exploratory study investigated the extent of former (last 2 years) and current staff exchange within and between nursing homes in The Netherlands. We relied on a questionnaire that was targeted towards nursing-home staff members who had contact with residents. We found that 17·9% and 12·4% of the nursing-home staff formerly (last 2 years) or currently worked in other healthcare institutes besides their job in the nursing home through which they were selected to participate in this study. Moreover, 39·7% of study participants worked on more than one ward. Our study shows that, in The Netherlands, nursing-home staff form a substantial number of links between wards within nursing homes and nursing homes are linked to a large network of healthcare institutes through their staff members potentially providing a pathway for MRSA transmission between nursing homes and throughout the country.

## INTRODUCTION

The prevention and control of methicillin-resistant *Staphylococcus aureus* (MRSA) is a public health priority within the European Union [1]. The lowest percentage of MRSA found in *Staphylococcus aureus* blood isolates (or ‘isolate prevalence’ of MRSA) in the 29 reporting European Union countries and two of the three European Economic Area countries (Norway and Iceland) is found in Norway, Sweden, and The Netherlands [2], which may be attributed to effective control measures of these countries and their prudent use of antibiotics [3]. Compared with the European mean blood isolate prevalence of MRSA in 2014 of 17·4%, the isolate prevalence in The Netherlands was notably low at 0·9% [2].

In 2014, the prevalence of MRSA carriage in the nursing-home population of The Netherlands was estimated to be 0·3% [4]. Despite this low prevalence, outbreaks in nursing homes are reported regularly [4]. Since 2011, The Netherlands has been subject to multiple MRSA *spa*-type 1081 (MRSA-t1081) outbreaks across the country [5]. All cases concerned carriage and hardly any infections were reported. Noteworthy for outbreaks involving this strain was the higher MRSA frequency in nursing homes compared to hospitals. While the spread of MRSA between hospitals is associated with inter-hospital patient transfers [6–8], the transfer of residents between nursing homes is uncommon in The Netherlands, raising questions on how MRSA-t1081 could spread through the nursing homes and across the country.

Only a few studies have addressed the spread of MRSA within and between nursing homes [9–12], one of which explicitly discussed staff exchange as a source of inter-institutional transmission [12]. Several studies reviewed in Albrich & Harbarth [13] demonstrated that employment on another ward or at another institution leads to intra- or inter-hospital MRSA transmission. Other studies provided both molecular and epidemiological evidence of MRSA transmission from asymptomatic healthcare workers to patients [13]. In most cases, however, the healthcare worker simply provided a mode of transfer, acting as a vector, and was not the main source of transmission [13]. While the risk factors associated with MRSA colonization in staff remain unclear, transmission of MRSA by hospital staff has been linked to poor infection control practices, such as inadequate handwashing [12, 13].

We hypothesized that staff exchange, and subsequent healthcare worker-to-patient transmission, plays a role in MRSA transmission between nursing homes in The Netherlands. In this study, we investigated the extent of staff exchange within and between nursing homes in The Netherlands.

## METHODS

### Data collection

Our study compared characteristics of Dutch nursing homes that had a recent MRSA-t1081 outbreak with nursing homes that had not. Twenty-five nursing homes were invited to participate in our study: eight were all the nursing homes that were implicated in the 2014/2015 MRSA-t1081 outbreak and were identified through the national MRSA surveillance programme administered by the Dutch National Institute of Public Health and the Environment (hereafter: ‘case nursing homes’); 17 nursing homes formed part of a recent Dutch study that included nursing homes with >50 beds for a point prevalence measurement of MRSA from October 2012 to July 2014 [4]. In these 17 nursing homes, MRSA was not detected from October 2012 to the start of the current study (hereafter: ‘control nursing homes’) [4].

Our exploratory study relied on a questionnaire to assess staff exchange in nursing homes in The Netherlands. We specifically sought information on the percentage of nursing-home staff that (1) were formerly (last 2 years) or currently employed at another healthcare institute (HCI) besides the nursing home through which they were selected to participate in this study (hereafter: ‘inter-institutional staff exchange’) and (2) normally worked on more than one ward in the nursing home through which they were selected to participate in this study (hereafter: ‘intra-institutional staff exchange’). The final questionnaire (see Supplementary Appendix 1, along with an English translation) consisted of eight questions (described in Table 1) and was targeted towards nursing-home staff members who had contact with residents.

**Table 1.** Operationalization of the concept ‘staff exchange’

Subconcepts	Indicator
Staff characteristics	Job type: *nursing staff; nurse; housekeeping; physiotherapist; hairdresser; other* Working hours per week Total years of employment
Staff exchange	In the nursing home through which you were selected to participate in this study, on how many wards do you usually work? Are you employed at a healthcare institute (HCI) besides the nursing home through which you were selected to participate in this study? [*yes/no*] If yes, select the type(s)* of HCI(s) and the number of each type. *Nursing home; residential home with nursing department; residential home without nursing department; rehabilitation institute; home care; hospital* List the name(s) of the(se) HCI(s) and how many hours per week you work at the(se) HCI(s) Besides the HCI(s) mentioned above, were you employed at another HCI in the past 2 years? If yes, select the type(s)* of HCI(s) and the number of each type. *Nursing home; residential home with nursing department; residential home without nursing department; rehabilitation institute; home care; hospital* List the name(s) of the(se) HCI(s) and how many hours per week you work at the(se) HCI(s)

* We provide a definition of the different types of HCIs in Supplementary Appendix 2.

Invitations to participate in the study were sent by mail to the 25 selected nursing homes. The nursing homes were asked to invite all their staff to participate in the questionnaire. The questionnaire was administered and responses were received from May to June 2015 using the online survey software questback.com.

### Data analysis

A descriptive analysis was conducted to provide an overview of staff member characteristics, separately for case and control nursing homes. We compared case and control nursing homes using an unadjusted *χ*^2^ test to assess significant differences between positions held by study participants, and the different types of HCIs in which participants worked in the past 2 years and were currently working. A *P* value <0·05 indicated a statistically significant result. An independent-sample *t* test with a significance level of 0·05 was used to demonstrate significant differences between the two groups for average hours worked and average employment duration. We relied on non-overlapping 95% confidence intervals (CIs) to indicate significant differences between the two groups in inter-institutional staff exchange. Differences in intra-institutional staff exchange were compared using CIs that were adjusted for clustering within nursing homes. All quantitative analyses were performed in R v. 3.1.0 [14].

## RESULTS

### Staff characteristics

Of the nursing homes invited to participate in our study, 75% (6/8) of the case nursing homes (173 staff members) and 53% (9/17) of the control nursing homes (301 staff members) responded and were included in our analysis (Table 2). Ten of the 15 nursing homes provided data on their total staff size, which ranged from 15 to 200 staff members. Within these nursing homes, the staff participation rate was 51·9% (154/297, range 45–73) in case nursing homes and 43·4% (269/619, range 29–67) in control nursing homes. The total staff participation rate in these nursing homes was 46·2% (423/916), which comprised 89·2% (423/474) of the staff included in our study. Study participants included nursing staff (63%), nurses (13%), physiotherapists (6%), nursing assistants (4%), elderly-care physicians (2%), and others (e.g. nutritionists and social workers). On average, participants worked 24·1 h per week and the number of years of employment in the current positions was 10·8 years. There was no statistical difference between the case and control groups in the numbers of hours worked and the number of years of employment; however, the distribution of positions held by the staff members participating in our study was significantly different between the case and control groups (Table 2). For example, compared to cases, the control group consisted of a larger percentage of nurses and a smaller percentage of nursing staff and nursing assistants (Table 2).

**Table 2.** Characteristics of nursing-home staff members who participated in our study

Staff characteristic	All participants	Participants working in control nursing homes	Participants working in case nursing homes	*P* value comparing significant differences between cases and controls
Total staff, *N*	474	301	173	
Nursing staff, *n* (%)	299 (63·1)	177 (58·8)	122 (70·5)	0·001 (*χ*^2^ test)
Nurse, *n* (%)	59 (12·4)	49 (16·3)	10 (5·8)	
Nursing assistant, *n* (%)	20 (4·2)	8 (2·7)	12 (6·9)	
Physician, *n* (%)	9 (1·9)	7 (2·3)	2 (1·2)	
(Physio)therapist, *n* (%)	30 (6·3)	17 (5·6)	13 (7·5)	
Housekeeping, *n* (%)	11 (2·3)	8 (2·7)	3 (1·7)	
Other, *n* (%)	46 (9·7)	35 (11·6)	11 (6·4)	
Working hours per week				0·805 (*t* test)
Mean (s.d.)	24·1 (8·8)	24·2 (9·1)	24·0 (8·2)	
Median (IQR)	24·0 (18·0–32·0)	24·0 (18·0–30·0)	24·0 (18·0–32·0)	
Time in position, years				0·989 (*t* test)
Mean (s.d.)	10·8 (10)	11·1 (10·2)	10·2 (9·8)	
Median (IQR)	7·0 (3·0–16·0)	8·0 (3·0–17·0)	7·0 (3·0–14·0)	

s.d., Standard deviation; IQR, interquartile range.

### Inter-institutional staff exchange

**Fig. 1.**
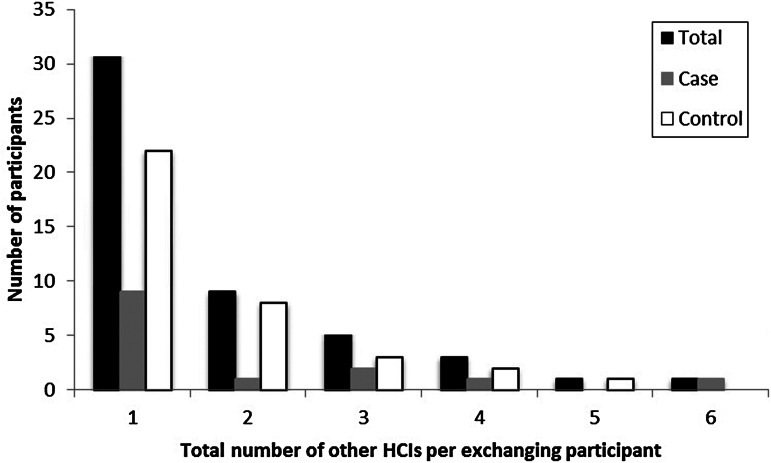
Distribution of the number of other healthcare institutes (HCIs) where participants are employed besides the job in the nursing home through which they were selected to participate in this study.

**Fig. 2.**
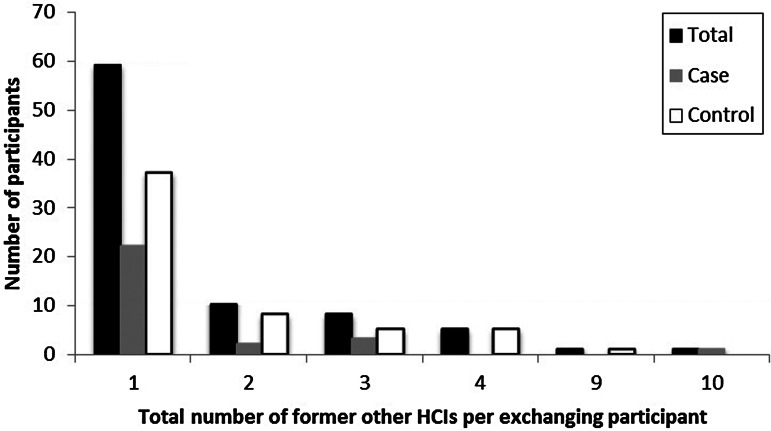
Distribution of the number of other healthcare institutes (HCIs) where participants were formerly (in the past 2 years) employed before the job in the nursing home through which they were selected to participate in this study.

In total, 12·4% (59/474) of participants were employed by at least one (but, for a couple, more than five) other HCI(s) simultaneously (Table 3, Fig. 1). Of the participants at case and control nursing homes, 8·7% and 14·6%, respectively, were working at more than one HCI concomitantly (Table 3). The 59 participants employed at more than one HCI reported forming 87 connections with other HCIs, where some participants may have been connected to the same other HCI. Of these other HCIs, most were nursing homes (63%) or residential care homes with nursing departments (15%). Within the previous 2 years, 17·9% (85/474) of the participants had been employed at a total of 142 HCIs other than where they were currently working (Tables 3 and 4). While most participants had been employed at only one or two other HCIs, two participants reported having previously worked at nine or more other HCIs (Fig. 2). The majority of the former employment was at other nursing homes (49%); however, many participants had also been employed at residential care homes (with and without nursing departments), home care, and hospitals (Table 5). While a higher percentage of participants in the control group participated in staff exchange than did the case group, the difference between the case and the control groups for all inter-institutional exchange characteristics (i.e. number and type of HCIs, for current and former employment) was statistically insignificant in all cases (Tables 3–5).

**Table 3.** Exchange within nursing homes (intra-institutional) and with other healthcare institutes (HCIs) (inter-institutional) of nursing-home staff members who participated in our study

	All participants		Participants working in control nursing homes		Participants working in case nursing homes	
Type of exchange	*n*	% (95% CI)	*n*	% (95% CI)	*n*	% (95% CI)
Employed at more than one HCI	59	12·4 (9·5–15·4)	44	14·6 (10·6–18·6)	15	8·7 (4·5–12·9)
Former (last 2 years) employment at other HCI(s)	85	17·9 (14·5–21·4)	57	18·9 (14·5–23·4)	28	16·2 (10·7–21·7)
Exchange with other wards	188	39·7 (35·4–44·1)*	133	44·2 (38·7–49·8)*	55	31·8 (25·3–39·1)*

CI, Confidence interval.

* 95% CI adjusted for clustering within nursing homes using the epiR package in R v. 3.1.0 [14, 15].

**Table 4.** Number and type of healthcare institutes where participants were formerly (in the past 2 years) employed before the job in the nursing home through which they were selected to participate in this study

Other former employment	Total	Participants working in control nursing homes	Participants working in case nursing homes
Total number of healthcare institutes, N*	142	97	45
Nursing home, *n* (%)	69 (48·6)	43 (44·3)	26 (57·8)
Residential care home with nursing department, *n* (%)	18 (12·7)	14 (14·4)	4 (8·9)
Residential care home without nursing department, *n* (%)	14 (9·9)	9 (9·3)	5 (11·1)
Home care, *n* (%)	15 (10·6)	10 (10·3)	5 (11·1)
Hospital, *n* (%)	8 (5·6)	8 (8·2)	0 (0)
Other/not known, *n* (%)	18 (12·7)	13 (13·4)	5 (11·1)

* *P* value from the unadjusted *χ*^2^ test comparing the six types between case and control nursing homes = 0·32.

**Table 5.** Number and type of healthcare institutes where participants work besides their job in the nursing home through which they were selected to participate in this study

Other current employment	Total	Participants working in control nursing homes	Participants working in case nursing homes
Number of jobs in healthcare institutes, N*	87	60	27
Nursing home, *n* (%)	55 (63·2)	35 (58·3)	20 (74·1)
Residential care home with nursing department, *n* (%)	13 (14·9)	11 (18·3)	2 (7·4)
Residential care home without nursing department, *n* (%)	3 (3·4)	3 (5·0)	0 (0)
Home care, *n* (%)	6 (6·9)	3 (5·0)	3 (11·1)
Hospital, *n* (%)	5 (5·7)	3 (5·0)	2 (7·4)
Other/not known, *n* (%)	5 (5·7)	5 (8·3)	0 (0)

* *P* value from the unadjusted *χ*^2^ test comparing the six types between case and control nursing homes = 0·22.

### Intra-institutional staff exchange

Overall, 39·7% of participants reported working on more than one ward within the nursing home through which they were selected to participate in this study. Of the participants employed at the case and control nursing homes, 31·8% and 44·2%, respectively, worked on more than one ward. Differences between the case and control nursing homes were not statistically significant (Table 3).

## DISCUSSION

To the best of our knowledge, this is the first study to **l**ook into staff exchange of nursing homes in The Netherlands and only one study has explored the extent of nursing-home staff exchange in other countries [12]. Sie *et al.* showed in their Norwegian study that 18·0% (95% CI 13·0–23·0) of nursing-home staff (41/228 from 42 nursing homes) worked at more than one HCI simultaneously [12]. Similarly, we found that 12·4% (95% CI 9·5–15·4) of the nursing-home staff worked in other HCIs besides their job in the nursing home through which they were selected to participate in this study. Moreover, we found that in the past 2 years 17·9% (95% CI 14·5–21·4) of the staff members had been employed at additional HCIs. In addition to inter-institutional staff exchange, we found that 39·7% of study participants worked on more than one ward.

Indications of inter-institutional MRSA transmission by new employees and through staff exchange are available in the literature [8, 13] and healthcare workers have been suggested as an MRSA transmission route between wards of a HCI [13]. Nevertheless, few studies have investigated the role of healthcare workers in transmission between nursing homes and between wards of a given nursing home. A causal relationship between staff exchange and MRSA transmission has yet to be demonstrated. In our study, we found no significant differences between the case and control nursing homes for both inter- and intra-institutional staff exchange. However, since our questionnaire assessed the current degree of staff exchange after the MRSA t1081 outbreaks had occurred, it is possible that nursing homes with a recent MRSA outbreak implemented new staff-exchange measures to curb transmission. This is a limitation of this study which may explain why we observed higher (although not statistically significantly higher) percentages of staff exchange in the control group compared to the case group.

For our study we invited all eight nursing homes implicated in the 2014/2015 MRSA t1081 outbreak to participate and selected controls from a previous study that had invited all nursing homes in The Netherlands with >50 beds to participate [4]. Despite our relatively high staff response rates (a strength of this study), the findings of our study may be subject to non-response bias between nursing homes and within nursing homes. Nursing homes in which there was an MRSA t1081 outbreak in 2014/2015 may have been more inclined to participate if measures were put in place to reduce transmission due to staff exchange. Nursing homes that were not implicated in an MRSA t1081 outbreak between 2013 and 2015 may have been less inclined to participate, in general. Within participating nursing homes, our response rate in 10 of the 15 nursing homes providing denominator data was 46·2%. This is likely an overestimate of the overall staff response rate since 33·3% of the nursing homes that did not provide data on the total staff size provided only 10·8% of the staff participants. Staff that work fewer hours than average at a nursing home participating in our study may have been less likely to submit a completed questionnaire than those who work more hours than average, and may be employed at more HCIs than staff who work longer hours at a participating nursing home (selection bias). Furthermore, our study did not collect data from interns who exchange more frequently between and within nursing homes and HCIs than staff members.

Despite these limitations, this study shows that, in The Netherlands, (1) nursing-home staff form a substantial number of links between wards within nursing homes, and (2) nursing homes are linked to a large network of HCIs through their staff members. This network may provide a pathway of MRSA transmission between nursing homes, although this was not observed to be implicated in this analysis. Additional studies are necessary to investigate the precise role of staff exchange on MRSA transmission within and between nursing homes. For example, a detailed investigation of an outbreak is needed to unravel the transmission tree and a modelling study would be helpful in investigating the likely impact of staff exchange on MRSA transmission.

## Supplementary material

For supplementary material accompanying this paper visit http://doi.org/10.1017/S0950268816002831. click here to view supplementary material
