# Supplementary material for: Staff exchange within and between nursing homes in The Netherlands and potential implications for MRSA transmission
Source: Epidemiol Infect. 2016 Dec 5;145(4):739–45. doi: 10.1017/S0950268816002831 (PMC5426333; doi:10.1017/S0950268816002831)
Supplement: Supplementary file 1 [file S0950268816002831sup001.docx]

**Epidemiology and Infection**

**Staff exchange within and between nursing homes in the Netherlands and implications for MRSA transmission**

R. D. van Gaalen, H. A. Hopman, A. Haenen, C. van den Dool

**SUPPLEMENTARY MATERIAL**

# Appendix 1 – Staff exchange questionnaire NB: an English translation is provided later in this section

**Wat is de naam van uw verpleeghuis?
___

Wat is uw functie?**

❑ Verpleegkundige

❑ Verzorgende

❑ Afdelingsassistent

❑ Huishoudelijk medewerker

❑ Fysiotherapeut

❑ Tandarts

❑ Anders, namelijk:

**Hoeveel uren in de week werkt u meestal bij dit verpleeghuis*?**

*met verpleeghuis wordt ook de verpleegafdeling van een verzorgingstehuis bedoeld.

**Hoe lang bent u al in dienst bij dit verpleeghuis*? (in jaren, indien u korter dan één jaar werkzaam bent bij dit verpleeghuis gelieve dit in maanden te noteren)**

*met verpleeghuis wordt ook de verpleegafdeling van een verzorgingstehuis bedoeld.

**Op hoeveel zorgeenheden* werkt u standaard?**

*gedefinieerd als functionele eenheid met een vaste groep personeelsleden die gezamenlijk 24-uurs verzorging regelen voor een vaste groep patiënten ,fysiek gescheiden van andere eenheden; ook vaak aangeduid als afdeling ,unit, etage, locatie, leefgroep, en dergelijke.

Indien bij u een zorgeenheid anders gedefinieerd is, gaarne toelichting.

**Werkt u naast dit verpleeghuis* nog bij een andere zorginstelling**?**

* hiermee wordt ook werken op de verpleegafdeling van het verzorgingstehuis bedoeld.

** hiermee wordt zowel werken op een andere locatie als het verlenen van zorg aan verzorgingstehuisbewoners op dezelfde of andere locatie bedoeld.

❑ Ja

❑ Nee

**Wat voor zorginstelling(en) is/zijn dit?**

|  | Verpleeghuis of verpleegafdeling binnen een verzorgingstehuis | Verzorgingstehuis met verpleegafdeling (waar u zorg verleent aan verzorgingstehuis-bewoners) | Verzorgingstehuis zonder verpleegafdeling | Thuiszorg | Revalidatie kliniek | Ziekenhuis |
| --- | --- | --- | --- | --- | --- | --- |
| 1 | ❑ | ❑ | ❑ | ❑ | ❑ | ❑ |
| 2 | ❑ | ❑ | ❑ | ❑ | ❑ | ❑ |
| 3 | ❑ | ❑ | ❑ | ❑ | ❑ | ❑ |
| 4 | ❑ | ❑ | ❑ | ❑ | ❑ | ❑ |
| 5 | ❑ | ❑ | ❑ | ❑ | ❑ | ❑ |
| 6 | ❑ | ❑ | ❑ | ❑ | ❑ | ❑ |

**Kunt u de naam/namen van de zorginstelling(en)hieronder invullen? En daarnaast hoeveel uur per week u hier werkzaam bent?**

**Heeft u in de afgelopen twee jaar naast bovengenoemde nog bij één of meerdere andere zorginstelling(en) gewerkt?**

❑ Ja

❑ Nee

**Wat voor zorginstelling(en) was/waren dit?**

|  | Verpleeghuis of verpleegafdeling binnen een verzorgingstehuis | Verzorgingstehuis met verpleegafdeling (waar u zorg verleent aan verzorgingstehuis-  bewoners) | Verzorgingstehuis zonder verpleegafdeling | Thuiszorg | Revalidatie kliniek | Ziekenhuis |
| --- | --- | --- | --- | --- | --- | --- |
| 1 | ❑ | ❑ | ❑ | ❑ | ❑ | ❑ |
| 2 | ❑ | ❑ | ❑ | ❑ | ❑ | ❑ |
| 3 | ❑ | ❑ | ❑ | ❑ | ❑ | ❑ |
| 4 | ❑ | ❑ | ❑ | ❑ | ❑ | ❑ |
| 5 | ❑ | ❑ | ❑ | ❑ | ❑ | ❑ |
| 6 | ❑ | ❑ | ❑ | ❑ | ❑ | ❑ |
| 7 | ❑ | ❑ | ❑ | ❑ | ❑ | ❑ |
| 8 | ❑ | ❑ | ❑ | ❑ | ❑ | ❑ |
| 9 | ❑ | ❑ | ❑ | ❑ | ❑ | ❑ |
| 10 | ❑ | ❑ | ❑ | ❑ | ❑ | ❑ |
|  |  |  |  |  |  |  |

**Kunt u de naam/namen van de zorginstelling(en) hieronder invullen? En daarnaast hoeveel uur per week u hier werkzaam was?**

**Mocht u nog opmerkingen hebben die voor dit onderzoek van belang kunnen zijn, zou u die dan hieronder willen invullen?**

**English translation of the original questionnaire**

**What is the name of your nursing home?
___

What is your position?**

❑ Nurse

❑ Nursing staff

❑ Nursing assistant

❑ Housekeeping

❑ Physiotherapist

❑ Dentist

❑ Other, namely:

**How many hours per week do you work at this nursing home*, on average?**

*this can also be a nursing department within a residential care home.

**How long have you been employed at this nursing home*? (in years, if less than one year, please indicate the number of months)**

*this can also be a nursing department within a residential care home.

**On how many wards* do you normally work?**

*defined as a functional unit, physically separated from other units, with a fixed staff, that provides 24-hour care to a fixed group of patients; these may also be referred to as department, unit, floor, location, community, or similar.

If the unit where you work is defined differently, please describe.

**Apart from this nursing home*, do you work at other health care institutions**?**

* this can also be a nursing department within a residential care home.

** this includes both working at another location and administering care for the residents of residential care homes at the same or another location

❑ Yes

❑ No

**What type of health care institutions are these?**

|  | Nursing home or nursing department within a residential care home | Residential care home with a nursing department (where you provide care to the residents of the residential care home) | Residential care home without a nursing department | Home care | Rehabilitation  clinic | Hospital |
| --- | --- | --- | --- | --- | --- | --- |
| **1** | **❑** | **❑** | **❑** | **❑** | **❑** | **❑** |
| 2 | ❑ | ❑ | ❑ | ❑ | ❑ | ❑ |
| 3 | ❑ | ❑ | ❑ | ❑ | ❑ | ❑ |
| 4 | ❑ | ❑ | ❑ | ❑ | ❑ | ❑ |
| 5 | ❑ | ❑ | ❑ | ❑ | ❑ | ❑ |
| 6 | ❑ | ❑ | ❑ | ❑ | ❑ | ❑ |

**In the space below, please list the name(s) of these health care institution(s) and the number of hours per week that you work there.**

**In the past two years, have you worked at other health care institutions besides those listed above?**

❑ Yes

❑ No

**What sort of health care institution(s) were these?**

|  | Nursing home or nursing department within a residential care home | Residential care home with a nursing department (where you provide care to the residents of the residential care home) | Residential care home without a nursing department | Home care | Rehabilitation  clinic | Hospital | |
| --- | --- | --- | --- | --- | --- | --- | --- |
| 1 | ❑ | ❑ | ❑ | ❑ | ❑ | ❑ |  |
| 2 | ❑ | ❑ | ❑ | ❑ | ❑ | ❑ |  |
| 3 | ❑ | ❑ | ❑ | ❑ | ❑ | ❑ |  |
| 4 | ❑ | ❑ | ❑ | ❑ | ❑ | ❑ |  |
| 5 | ❑ | ❑ | ❑ | ❑ | ❑ | ❑ |  |
| 6 | ❑ | ❑ | ❑ | ❑ | ❑ | ❑ |  |
| 7 | ❑ | ❑ | ❑ | ❑ | ❑ | ❑ | |
| 8 | ❑ | ❑ | ❑ | ❑ | ❑ | ❑ | |
| 9 | ❑ | ❑ | ❑ | ❑ | ❑ | ❑ | |
| 10 | ❑ | ❑ | ❑ | ❑ | ❑ | ❑ | |
|  |  |  |  |  |  |  |  |

**In the space below please list the name(s) of these health care institution(s) and the number of hours per week that you work there.**

**In the space below, please provide any comments that could be relevant for this study.**

**Appendix 2 – Definition of the different types of HCIs included in this study**

In the Netherlands, several long term care environments exist that cover a range of care, rated from ZZP4 to ZZP10, with ZZP10 being the level of care provided to patients with the most severe illnesses (VWS, 2015). Patients with a ZZP4 indication (formerly also ZZP3) receive care in a residential care home (assisted living). Patients with a ZZP5-ZZP8 indication receive care in a nursing home or in a nursing home department within a residential care home. Patients with a ZZP5 or ZZP7 indication receive (intense) psychogeriatric care; those with ZZP6 or ZZP8 receive somatic care. Rehabilitation clinics or rehabilitation departments take care of patients with ZZP9. Patients with ZZP10 receive palliative care which is offered in separate institutions or departments (hospices) and are not included in this study.

**Reference**

Ministerie van Volksgezondheid, Welzijn en Sport (VWS), Regeling Langdurige zorg. Bijlage A. van artikel 2.1. 2015
